# The Role of the ECM in Lung Cancer Dormancy and Outgrowth

**DOI:** 10.3389/fonc.2020.01766

**Published:** 2020-09-11

**Authors:** Amelia L. Parker, Thomas R. Cox

**Affiliations:** ^1^Garvan Institute of Medical Research, Darlinghurst, NSW, Australia; ^2^St Vincent’s Clinical School, UNSW Sydney, Darlinghurst, NSW, Australia

**Keywords:** extracellular matrix, lung cancer, dormancy, metastasis, matrix-targeting

## Abstract

The dissemination of tumor cells to local and distant sites presents a significant challenge in the clinical management of many solid tumors. These cells may remain dormant for months or years before overt metastases are re-awakened. The components of the extracellular matrix, their posttranslational modifications and their associated factors provide mechanical, physical and chemical cues to these disseminated tumor cells. These cues regulate the proliferative and survival capacity of these cells and lay the foundation for their engraftment and colonization. Crosstalk between tumor cells, stromal and immune cells within primary and secondary sites is fundamental to extracellular matrix remodeling that feeds back to regulate tumor cell dormancy and outgrowth. This review will examine the role of the extracellular matrix and its associated factors in establishing a fertile soil from which individual tumor cells and micrometastases establish primary and secondary tumors. We will focus on the role of the lung extracellular matrix in providing the architectural support for local metastases in lung cancer, and distant metastases in many solid tumors. This review will define how the matrix and matrix associated components are collectively regulated by lung epithelial cells, fibroblasts and resident immune cells to orchestrate tumor dormancy and outgrowth in the lung. Recent advances in targeting these lung-resident tumor cell subpopulations to prevent metastatic disease will be discussed. The development of novel matrix-targeted strategies have the potential to significantly reduce the burden of metastatic disease in lung and other solid tumors and significantly improve patient outcome in these diseases.

## Lung Cancer Dynamics and Dormancy

Lung cancer remains the leading cause of cancer death worldwide (1). Non-small cell lung cancer (NSCLC), which consists of adenocarcinoma, squamous cell carcinoma and large cell carcinoma, accounts for more than 80% of all lung cancer cases (2). Adenocarcinoma and squamous carcinoma are the predominant NSCLC subtypes. Large cell carcinoma represents less than 5% of NSCLC cases and is diagnosed when the tumor cannot be identified by conventional adenocarcinoma and squamous markers. Large cell NSCLC is increasingly viewed as an undifferentiated form of squamous carcinoma and adenocarcinoma rather than a distinct histological subtype, as more sophisticated molecular characterization of these tumors identifies features of the adenocarcinoma or squamous carcinoma subtypes within these tumors. Adenocarcinoma and squamous carcinoma have distinct anatomical growth patterns, with adenocarcinoma and squamous carcinoma developing in the distal and central lung, respectively. NSCLC subtypes are believed to arise from different cells of origin in a context-dependent manner (3) although the etiology of non-small cell lung cancer subtypes remains to be precisely defined.

Effective management of NSCLC is hampered by the late presentation of the disease, when metastatic foci are typically already established. The major sites of non-small cell lung cancer metastasis are the brain (12–47%), bone (16–39%), liver (7–22%), intrapulmonary (to contralateral or ipsilateral lobes, 11–26%), pleura (10–13%), thoracic lymph node (29%) and adrenal gland (6–15%) (4–7). The specific organ tropism of lung cancer cells for these secondary sites partly depends on the histology and genomic profile of the primary tumor (8, 9). While the metastatic profile of adenocarcinoma and squamous carcinoma are similar, adenocarcinoma has a higher incidence of bone metastases and intrapulmonary metastases than squamous carcinoma (5, 10), suggesting that adenocarcinoma and squamous carcinoma cells, either intrinsically or through their interaction with their tumor microenvironment, are differentially programmed with regards to metastatic propensity and organ tropism. This review will unravel the current understanding of how the extracellular matrix of the primary lung tumor and secondary sites regulate the formation of metastatic foci in non-small cell lung cancer.

Aided by improved detection modalities (11, 12), lung cancer is increasingly being diagnosed during the early stages of progression, where curative-intent surgical resection is the front line therapy and is associated with significantly greater 5 year survival than disseminated disease. However, disease recurrence is prevalent even among early stage (stage I and II) NSCLC patients, with 30–60% of early stage patients with margin-negative resected lung cancer developing local or distant disease recurrence (13, 14). NSCLC has a relatively high rate of synchronous (25%) and metachronous (2% per year) tumors. These tumors may develop from multiple independent tumor initiating events or through contralateral or ipsilateral metastasis of a primary lung tumor, making it difficult to determine the true rate of intrapulmonary metastasis in NSCLC (15, 16). These etiologies are distinct processes, with the former occurring when multiple tumors are initiated independently in the lung but emerge metachronously due to differential reawakening of the dormant transformed cells *in situ*. Conversely, the latter occurs *via* a conventional metastatic process whereby cells disseminate away from the primary tumor to colonize a niche within the lung that is anatomically distinct from the primary tumor site.

Emerging evidence from DNA sequencing studies mapping the clonal evolution of lung tumors is providing unprecedented insight into the dynamics of lung tumor outgrowth, as well as distinguishing between tumors arising from intrapulmonary metastases or from independent transformation events. Correlations between multiregional tumor sequencing and smoking-associated behavior suggest that driver gene mutations occur several decades prior to cancer diagnosis (17) and therefore that primary and secondary NSCLC tumors are likely to undergo some period of dormancy before becoming re-awakened. Furthermore, intrapulmonary metastases are associated with a longer latency than distant metastases, commonly re-emerging more than 5 years following surgery (13). In addition to genetic changes, widespread and dispersed changes in the structure and composition of the lung ECM as well as the transcriptional profile of normal bronchial epithelia in smokers and lung cancer patients are thought to represent a field of cancerization that promotes tumor initiation and regulates the dissemination of lung tumor cells from the primary site (18–22). Similarly, the severe extracellular matrix remodeling in chronic lung diseases such as chronic obstructive pulmonary disorder (COPD) and idiopathic pulmonary fibrosis (IPF), which are associated with an increased risk of lung cancer development, may also contribute to this field effect (23, 24). Although the mechanisms underlying these clinical associations remain unclear, these associations support the notion that the extracellular matrix is an important regulator of NSCLC etiology.

The mechanisms that drive the dormancy and reawakening of lung cancer cells both within the lung and in other secondary organs remain to be precisely defined, however, there is a clear tissue tropism to the induction, maintenance and re-awakening of tumor cell dormancy that occurs in a cancer type-dependent manner (25, 26). The extracellular matrix is well recognized as a regulator of cellular proliferation and differentiation. Studies in other cancers have revealed mechanisms by which the matrix regulates this dormancy and the outgrowth of metastases, and these molecular alterations are also seen in lung cancer. As such, valuable insights into the dormancy and metastatic behavior of primary lung tumors come from studies of the metastatic colonization of the lung by non-pulmonary cancer cells, as well as studies of both primary and metastatic lung cancer. The importance of the extracellular matrix in regulating dormancy and re-activation is emerging as an important area of research, and a resource from which novel therapies targeting metastasis are being developed. This review addresses our current understanding of the role of the extracellular matrix in regulating the dormancy and emergence of both primary and secondary lung tumors.

## The Role of the ECM in Primary and Secondary Dormancy Dynamics

The accredited model of tumor dynamics is that disseminated cancer cells interact with both local and distal stromal and immunological cells through systemic and local signaling to establish niches that support the engraftment and survival of cancer cells. The seed and soil hypothesis states that for successful metastasis to occur, disseminated tumor cells must be compatible with the secondary niches that they go on to colonize. Disseminated tumor cells may be maintained in these metastatic niches in a dormant or quiescent state, and cells that are compatible with their new environment may later re-enter the cell cycle and start proliferating to form metastatic foci in response to intrinsic and microenvironmental cues (27). The extracellular matrix and associated factors engage with cell surface receptors to regulate intracellular signaling programs that control this exit and re-entry to the cell cycle in a context- and cell type- dependent manner. An emerging understanding of how these processes are co-ordinately regulated by the tumor microenvironment is challenging claims that this quiescence occurs stochastically (28).

**FIGURE 1:**
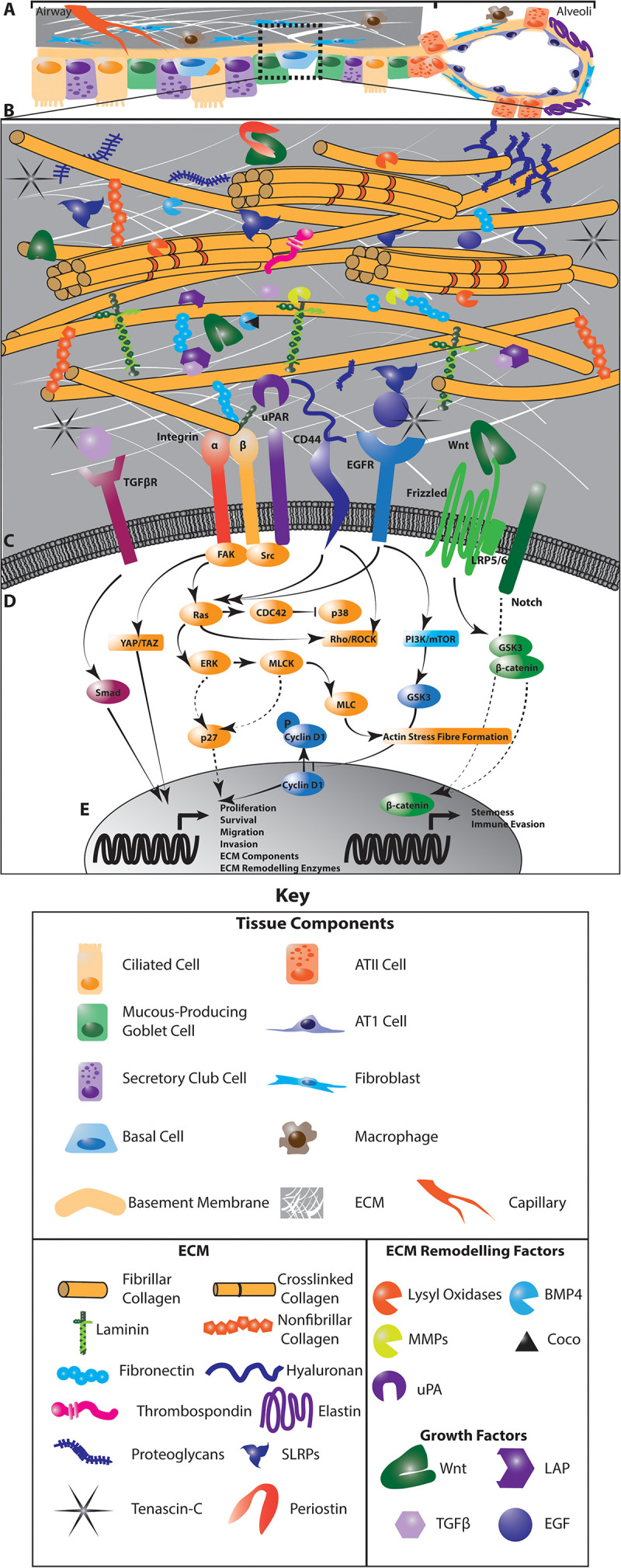
The interaction of cancer cells with the extracellular matrix (ECM) in the lung regulates diverse intracellular signaling pathways to regulate cancer cell dormancy, proliferation and metastases in NSCLC. Multiple cell types within the lung generate and remodel the extracellular matrix **(A)**. The composition of the ECM differs from the central airways and peripheral parenchyma where alveoli enable gas exchange, and is highly dysregulated in NSCLC **(A)**. This ECM is composed of matrisomal proteins, factors that associate with these proteins as well as enzymes involved in remodeling this matrix **(B)**. The posttranslational modification of ECM proteins and their interaction with each other ECM components determines the biomechanics of the tissue. ECM remodeling also releases growth factors and reveals cryptic sites within ECM components that are recognized by cells. Receptors on the cell membrane directly interact with specific ECM components and secreted factors **(C)** to activate diverse intracellular signaling programs that regulate the proliferative and metastatic characteristics of cancer cells **(D)**. These signaling programs drive the transcription of cell cycle regulators, stem cell markers and genes involved in EMT, migration, invasion and immune activation to regulate tumor dynamics **(E)**. In addition, these signaling programs activate the expression of ECM genes and ECM remodeling enzymes to drive further evolution of the tumor microenvironment **(E)**. Dotted lines indicate indirect interactions. ATII, Type II pneumocyte; ATI, Type I pneumocyte.

The extracellular matrix of the lung incorporates a diverse group of core matrisomal proteins that form the structural basis of the tissue, together with the enzymes responsible for remodeling and processing these molecules, as well as the soluble factors that are associated with this matrix (29) (Figure 1). Together, these matrisomal proteins function as a dynamic network of structural and signaling effectors that undergo constant remodeling. The function of the central airways and distal parenchyma are profoundly shaped by regional differences in the extracellular matrix composition and architecture of these compartments (Figure 1A). Associations between the ECM composition of NSCLC tumors and the risk of disease recurrence (30), points to a role for the extracellular matrix in regulating cancer cell dissemination, dormancy and outgrowth in intrapulmonary and distant metastases.

While mechanisms of dormancy appear strongly cell-type and context dependent, these pathways largely converge on mitogenic and stress response signaling pathways. In particular, the TGFβ/BMP, FAK, Src, uPA, EGFR, and integrin signaling pathways are integrated with LKB1/AMPK, PI3K and metabolic signaling to modulate ERK/p38, JNK, cyclin and downstream cell cycle regulators to control the entry and exit of cells from the cell cycle (27, 31, 32) (Figure 1D). For example, integrin receptor and uPAR activation converge on the ERK/p38 pathway where low ERK1/2 activity coupled with high p38 activity drives a dormant phenotype (33) through transcriptional regulation that is associated with poor progression-free survival in numerous cancers (34) (Figure 1E). Crosstalk between integrin signaling and the EGFR and uPAR pathways, which intersect with the downstream effectors of integrin receptors, including FAK, also regulate dormancy by these same p38 and p27-dependent pathways, as well as through Akt signaling cyclin D1 activation (35, 36). In addition, stem cell programs characterized by pro-survival, quiescent signaling also support dormancy and metastasis. For example, cues from the extracellular matrix interact with Wnt and Notch signaling to mediate these quiescent phenotypes (Figures 1D,E). How these pathways operate specifically in lung cancer remains less clear than in other well-studied cancer types such as breast cancer (37). However, concordant transcriptional signatures uncovered in breast metastases within the lung and models of aggressive metastatic lung cancer (38) suggest that common mechanisms involving the ECM may operate across cancer types. Understanding the effect of the extracellular matrix in orchestrating these intersecting dormancy signaling pathways lays the foundation for developing therapeutic approaches to improve lung cancer treatment.

### The Core Matrisome

The extracellular matrix composition of the central lung is dominated by fibrillar collagens (primarily collagen types I and II), while the interstitial ECM of the alveoli in the distal lung is a relaxed network of mainly type I and II collagens and elastin (Figure 1A). Compared with normal lung tissue, primary lung tumors display significant changes in the core matrisomal proteins that maintain the structural and mechanical features of the tissue. Due to their different anatomical locations, adenocarcinoma and squamous cancer cells are exposed to different extracellular matrix environments (Figure 1A), which likely shape the evolution of these tumor types and thus contribute to differences in etiology. While direct comparisons of the ECM landscape of adenocarcinoma and squamous carcinoma with respect to peripheral and central ECM composition in healthy lung have not yet been performed, consideration of the ECM composition in different lung anatomical compartments will be important in identifying shared ECM remodeling programs that generally contribute to lung tumorigenesis across multiple subtypes. Even within these broad histological subtypes, the extracellular matrix landscape differs between distinct molecular subclasses of adenocarcinoma and squamous carcinomas (39). Whilst oncogenic driver mutations are known to contribute to some of this heterogeneity in both the primary and secondary NSCLC tumors, it is now clear that our understanding should include extracellular matrix components that also modulate tumor heterogeneity. Precisely how these anatomical differences in the lung affect non-small cell lung cancer dormancy dynamics, and indeed whether they play a role in the preferential colonization of the lung by non-pulmonary tumor cells, remains unclear. Emerging evidence does, however, point strongly to roles for the core matrisome composition and architecture in regulating the dormancy and outgrowth of cancer cells within the lung as well as at other common NSCLC secondary sites (Table 1).

**TABLE 1:** Summary of functional studies identifying a direct role for ECM components in Dormancy and outgrowth in solid cancers affecting the lung.

**ECM category**	**ECM component**	**Role**	**Experimental system**
*Collagens*	Collagens	Increased fibrillar collagen levels promotes proliferation, metastasis and outgrowth Increased type IV collagen, decreased type III and type XVIII collagens promote liver metastasis and outgrowth	*In vivo* models and human biospecimens (40) *In vivo* models and human biospecimens (43)
*Glycoproteins*	Laminin	Laminin-cleavage promotes dormant re-awakening	Metastatic breast cancer model (61, 62)
	Fibronectin	Increased fibronectin promotes lung colonization and outgrowth, NSCLC proliferation and invasion	Metastatic lung cancer mouse model (66) Metastatic breast cancer mouse model (68) Biospecimens of metastatic human breast cancer (67) *In vitro* NSCLC models (70, 71)
	Tenascin-C	Promotes adenocarcinoma metastasis Promotes lung colonization	*In vivo* and human biospecimens (40) *In vivo* breast cancer model(80) *In vivo* breast cancer model (84)
	Periostin	High expression is associated with poor prognosis High expression supports lung colonization and outgrowth	Human biospecimens (85–88) *In vivo* breast cancer model (85)
	Thrombospondin	High expression of thrombospondin-2 supports lung colonization and metastasis of primary NSCLC High thrombospondin-1 expression inhibits tumorigenesis	*in vivo* breast cancer model and human biospecimens (93) human NSCLC biospecimens (95) *in vitro* and *in vitro* models (94) *in vivo* NSCLC model (98)
	Osteopontin	High expression is associated with poor survival High expression promotes invasion	Human NSCLC biospecimens (102, 103) *In vitro* models (104, 105)
*Proteoglycans and Glycosaminoglycans*	Versican	High expression is associated with poor survival	Human NSCLC biospecimens (111)
	Hyaluronan	High expression of hyaluronan or CD44 promotes tumor recurrence High expression promotes tumor proliferation and outgrowth	Human NSCLC biospecimens (111, 117) *in vitro* models, *in vivo* models and human biospecimens (39, 115)

#### Collagens

Collagens are a significant component of the lung ECM and altered collagen composition and structure are known to regulate cancer cell dormancy. Primary lung tumors of both the adenocarcinoma and squamous carcinoma subtypes have increased fibrillar collagen deposition and an altered collagen architecture that is consistent with a fibrotic response (40) (Figure 1B). In breast cancer models, increased fibrillar collagen activates β1- and β4- integrin signaling to promote the colonization and proliferation of metastatic foci in the lungs (41) as well as the re-awakening of disseminated dormant tumors cells (42), and as such, similar mechanisms are expected to operate in primary NSCLC tumors. Increased expression of type IV collagen, and decreased expression of collagen III and collagen XVIII are associated with NSCLC liver metastases, where they activate pro-survival α2β1-integrin signaling to sustain NSCLC cancer cells within the liver (43), although the mechanisms driving these changes are unknown. In primary lung adenocarcinoma, fibrosis seen in mediastinal lymph node metastases resembles that of the primary lung adenocarcinoma (40), suggesting that either intrinsic features of the primary cancer cells promote a fibrotic environment at secondary sites through crosstalk with stromal cells, or that aberrant fibrosis at secondary sites may promote overt outgrowth of the disseminated cells that drain there.

Whilst changes in the composition and architecture are the major collagen alterations in lung tumors, mutations in collagen type V alpha 2 chain (COL5A2) and collagen type II alpha I chain (COL2A1) genes have also been detected in subclonal secondary lung tumors following genomic doubling events in both adenocarcinoma and squamous carcinoma (44). While somatic mutations in extracellular matrix components are considered rare, and of lesser influence on the tumor extracellular matrix landscape than dynamic compositional and architectural changes, the effect of these collagen mutations on the extracellular matrix architecture and function remain to be investigated.

The collagen composition of tissues is partly determined by the activity of proteases that process nascent collagen molecules for assembly into the 3D environment. Bone Morphogenetic Protein 1 (BMP1) and members of the A Disintegrin and Metalloproteinase with Thrombospondin motifs (ADAMTS) family, which are differentially expressed in NSCLC tumors, are required to cleave the N- and C-termini of the pro-collagen peptide to enable collagen fibrillogenesis and deposition (45). It is not clear yet how altered expression of these proteases within the primary tumor might influence the increased deposition of fibrillar collagens in NSCLC. In addition to changes in the collagen composition, the fibrillar collagen architecture is altered in NSCLC, becoming more disordered and fragmented at the submicron scale but more aligned at the macro scale compared with normal lung tissue (46). The collagen architecture is regulated at these different length scales by collagen molecules binding to fibronectin, small leucine-rich proteoglycans and Fibril Associated Collagens with Interrupted Triple Helices (FACIT collagens) to nucleate the collagen network (47) as well as the activity of remodeling enzymes (see ECM Remodeling Factors) and cellular traction forces generated by resident cells. However, the mechanisms governing these broad architectural changes in the fibrillar collagen network in lung cancer remain unclear. Aligned collagen fibers seen in higher stage lung tumors would be expected to generate anisotropic biomechanics that have been shown to provide contact guidance cues to regulate cell shape and promote migration in breast cancer models (48). However, how the discrete collagen architecture in the central airways and distal lung contribute to the metastatic behavior of lung cancer cells remains to be investigated.

Collagens, as well as laminin, fibronectin, thrombospondin, osteopontin and other core matrisomal proteins mediate their effects on dormancy by acting as ligands for integrin receptors to activate intracellular proliferation pathways. Integrin receptors are heterodimers of α and β chains that bind to a variety of extracellular matrix molecules, and are the main cellular receptor for collagens. Integrin receptors are capable of bidirectional signaling across the plasma membrane. Intracellular signals regulate the conformation and ligand affinity of the extracellular domain while also mediating intracellular cytoskeletal signaling (35). For example, activation of β1-integrin has been shown to lead to activation of Src and subsequently FAK and ERK signaling via Ras to regulate dormancy (Figure 1D). The subsequent activation of MLCK in this signaling cascade also regulates actin dynamics and stress fiber formation (42), driving the translocation of p27 into the cytoplasm to initiate cell proliferation and re-entry of dormant cells into the cell cycle (49). Simultaneously, integrin receptor engagement activates CDC42, which inhibits p38 expression, thus resulting in a high ERK/low p38 profile that supports continued cell proliferation. Conversely, loss of receptor engagement drives a low ERK/high p38 profile that favors tumor cell dormancy, while negatively feeding back onto ERK activation to inhibit cell proliferation (50). Integrin signaling also has significant crosstalk with growth factor signaling. For example, EGFR signaling and activated integrin receptors can induce ligand-independent activation of EGFR signaling (51) (Figure 1D). Whether activating EGFR mutations, found in 15% of adenocarcinoma patients (52) directly perturb integrin signaling responses in NSCLC remains to be seen.

This extensive crosstalk between signaling pathways downstream of integrin receptors generates a network of diverse intracellular signaling pathways that are finely tuned to respond to heterogeneity within the extracellular matrix environment. Specific pairs of α- and β-integrin receptors have different affinities for extracellular matrix components, and enact different downstream signaling events. This creates a signaling program that can be fine tuned to interpret and respond to the subtle shifts in the extracellular environment (53). The collagen-binding integrin subunits β1 and α3 (which also bind fibronectin, laminin, and thrombospondin), together with the α5- (binds fibronectin), β3- (binds fibronectin, von-willebrand factor, and thrombospondin) and β4- (binds laminin) integrin receptors have been shown to be upregulated or functionally activated by their respective ligands within the lung niche to promote the proliferation and metastatic potential of cancer cells (42, 54–59). In this way, the collagen composition and presence of other integrin ligands in the lung can act as a switch between the dormant and proliferative state (49).

#### Glycoproteins

Glycoproteins are proteins that are covalently modified with carbohydrate groups. They have diverse functions within the primary tumor, pre-metastatic and metastatic niches including the regulation of dormancy and outgrowth. Below we will discuss the role of some of the most well studied glycoproteins involved in primary and secondary lung tumors.

##### Laminin

Laminin is a heterotrimeric glycoprotein that acts as an important component of the basement membrane in the lung as well as in other tissues (29). Basement membrane ECM components such as laminin have been shown to play an important role in regulating the cellular proliferative response in both health and disease (Figure 1A). Importantly, they are widely understood to promote tumor cell dormancy in non-pulmonary tumors (60).

The laminin-111 isoform in the lung basement membrane is known to regulate the dormancy of breast cancer cells colonizing the lung (61). Albrengues and colleagues found that laminin-111 digestion by neutrophil-derived matrix metalloproteinase-9 (MMP9) and elastase reveals cryptic sites that in turn activate β1-integrin signaling to re-awaken dormant breast cancer cells in the lung (61). This builds upon observations that MMP9 digestion of laminin-111 drives a loss of cell polarity and tumor growth in primary breast cancer models (62). Furthermore, oncogene-driven hyperactive Raf/MEK/ERK signaling induces MMP9 expression in breast cancer cells (62), suggesting that oncogenic features of the cancer cells may accelerate ECM remodeling-dependent mechanisms of reawakening. It is not known if these mechanisms also operate in primary lung tumors or intrapulmonary metastases derived from lung tumors, however, further investigations are warranted since neutrophil infiltration and Raf/MEK/ERK hyperactivation are both features of NSCLC tumors (63, 64).

##### Fibronectin

The glycoprotein fibronectin directly interacts with multiple extracellular matrix proteins as well as integrin receptors α3, α5, αV, α8, β1, and β3, which together regulate the structure of fibronectin fibrils, cell-ECM engagement and activate pro-proliferative intracellular signaling programs. Under cellular contraction, tensile forces applied to fibronectin reveal cryptic sites that bind to other fibronectin fibers, inducing fibronectin fibril formation that further stretches the fibers (65). These stretched fibronectin fibers act as binding sites for collagen fibers to regulate the fibrillar collagen network architecture. Fibronectin is widely implicated in regulating tumor cell dormancy in breast cancer and melanoma metastasis to the lung by regulating the architecture of surrounding ECM proteins, as well as by direct engagement of integrin receptors (66, 67). Growing evidence suggests that fibronectin may also play a similar role in primary lung cancer progression as well as intrapulmonary metastases.

Fibronectin expression is increased in primary lung tumor sites and at pre-metastatic sites of breast cancer metastasis to the lung (40, 66, 67). In breast cancer models, fibronectin is expressed by fibroblasts in the pre-metastatic niche due to the recruitment and activation of hematopoietic progenitors recruited to the lungs (66, 68). β1-integrin receptors on colonizing cancer cells are then activated upon fibronectin binding. This in turn activates MLCK and actin stress fiber formation that switches breast cancer cells from dormancy to proliferation (49). In primary NSCLC tumors, fibronectin-mediated β1-integrin activation also results in PI3K/Akt and FAK activation to drive pro-proliferative and pro-invasive signaling (69–71). To date, it is not yet clear if the arrangement of fibronectin fibers is significantly altered in NSCLC to regulate focal adhesion formation and FAK activation in a similar manner (72).

In addition to its direct stimulation of integrin signaling, fibronectin can indirectly influence growth factor signaling and cell proliferation by interacting with Bone Morphogenetic Protein-1 (BMP-1), Hepatocyte Growth Factor (HGF), Fibroblast Growth Factor 2 (FGF-2), Platelet-derived Growth Factor (PDGF) and latent TGFβ, although this is yet to be demonstrated specifically in NSCLC tumors (73). It is by these manifold mechanisms affecting cellular proliferation and migration that fibronectin is assumed to regulate lung tumor progression and metastasis. However, the role of this ECM protein in regulating gap junction formation in type II pneumocytes in healthy lung tissue (74, 75) suggests that fibronectin may also influence lung adenocarcinoma tumorigenesis by contributing to altered epithelial structure, cell polarity and potentially self renewal programs in this histological subtype.

##### Tenascin-C

Tenascin-C is a large multi-domain glycoprotein that undergoes extensive post-transcriptional and post-translational regulation. It is highly expressed in lung development during branching morphogenesis and alveolarization. However, in adult lungs it is only transiently expressed in response to injury (76). Loss of tenascin-C during lung development alters TGFβ signaling, reduces fibroblast to myofibroblast transformation and increases collagen deposition in small airways (76). Conversely, in response to injury, Tenascin-C stimulates TGFβ responsiveness to promote collagen deposition and fibrosis (77). Tenascin-C, like its binding partner fibronectin, is significantly upregulated in fibrotic lungs and in lung adenocarcinoma (40) raising the notion of a potentially overlapping mechanistic contribution to these disease processes. Tenascin-C transcription is also repressed by the canonical lung adenocarcinoma transcription factor Nkx2-1 (40). As adenocarcinoma progresses, Nkx2-1 expression decreases, releasing the suppression of Tenascin-C expression, which is thought to feed into accelerating tumor progression and metastasis (40).

Tenascin-C is also a ligand for β1-containing integrin receptors and is known to activate pro-proliferative integrin signaling. Together with its close association and interactions with fibronectin, its engagement with integrin receptors is a major mechanism by which this glycoprotein regulates tumor dynamics. Tenascin-C also contains EGF-like domains which can potentially directly activate EGFR signaling (78). While evidence that it directly activates EGFR signaling in NSCLC tumors is currently lacking, it remains possible that Tenascin-C may mediate some of its effects through this mechanism in EGFR wild type tumors. Tenascin-C also binds a number of pleiotropic growth factors, including TGFβ, PDGF, FGF and insulin-like growth factor (IGF) family members, and therefore likely plays an important role in regulating growth factor availability and proliferative signaling within the lung environment (79). Importantly, the role of tenascin-C as a regulator of TGFβ signaling appears to be central to its effects on dormancy in breast cancer cells colonizing the lungs and parallels may be drawn to primary lung tumors or intrapulmonary metastases. Following the dissemination of single breast cancer cells to the lungs, TGFβ signaling is downregulated in dormant cells, switching them into a pro-proliferative phenotype that establishes macrometastatic foci (80). This switch from dormancy to proliferation is determined by TGFβ availability, which is tightly regulated. TGFβ is sequestered to the matrix and remains unavailable by its complexation with the latency associated peptide, which is itself bound to integrin receptors, and latent TGFβ binding proteins, which are tethered to fibronectin, fibrillins, fibulins, proteoglycans and other fibrillar ECM components (73, 81, 82) (Figure 1B). This complex holds TGFβ in an inactive state, while its release from these complexes frees TGFβ to bind TGFβ receptors and activate pro-dormancy signaling. By interacting with TGFβ (79), tenascin-C sequesters TGFβ in an inactive state to prevent the induction of pro-dormancy cellular reprogramming and thereby enable proliferative signaling (83) (Figure 1E), although it is not clear if this is the central mechanism by which this ECM component contributes to aggressive NSCLC.

High expression of Tenascin-C in breast cancer is associated with increased risk of lung metastases, suggesting that it affects more than proliferative signaling in cancer cells. Here Tenascin-C acts in an autocrine manner on disseminated cancer cells to support their self-renewal, survival and lung colonization characteristics. Tenascin-C-mediated breast cancer cell reprogramming inhibits JAK2-STAT5 signaling to enhance MSI1 expression and drive pro-metastatic NOTCH signaling (84) (Figures 1C,D). Whether these interactions occur in subpopulations of cancer cells or equivalently across all cancer cells remains to be determined. As larger metastatic foci form in the lungs, Tenascin-C is increasingly expressed by activated fibroblasts in the metastatic niche (84). How this switch from cancer to fibroblast-mediated expression occurs, and whether these same mechanisms occur in primary and intrapulmonary secondary lung tumors remains unclear. Similar mechanisms observed in bone-tropic breast cancer metastases (84) suggests that Tenascin-C also supports metastatic outgrowth in bone metastases and may contribute to the metastasis of lung cancer cells to the bone microenvironment. Taken together, Tenascin-C appears to play a role in metastatic colonization at multiple sites, both those naturally rich in Tenascin-C and also in tissues that are activated to upregulate Tenascin-C in response to the presence of cancer cells. Further studies are warranted to dissect the mechanisms by which Tenascin-C affects the progression and dissemination of lung tumor cells, with particular attention to where these mechanisms converge with and diverge from its more established role in breast cancer metastasis.

##### Periostin

Periostin is a secreted glycoprotein that has also been shown to induce awakening of dormant cancer cells within the lung. High expression of periostin, as well as the aberrant expression of alternatively spliced isoforms of this gene, are also associated with poor prognosis in primary lung cancer (85–88). In metastatic breast cancer models, TGFβ2 and TGFβ3 expressed by disseminated tumor cells induce periostin expression in lung fibroblasts and endothelial tip cells (85, 89). Periostin within the lung environment directly interacts with Wnt agonists Wnt1 and Wnt3a to potentiate Wnt signaling and promote the stemness characteristics of disseminated breast cancer cells (85). This supports the survival of these cells and their self-renewal capacity in initiating secondary tumors. Together with its interaction with core matrisomal proteins (Tenascin C, fibronectin, and type I collagen) and ECM remodeling proteins such as lysyl oxidases, the high expression of periostin in lung cancer contributes to the structural and functional ECM network within lung tissue that enhances the metastatic potential of cancer cells (90). Importantly, in breast cancer metastasis models, periostin-mediated tumor initiation is specific to the lung microenvironment, since it does not affect the progression of primary breast tumors (85). For this reason, the pro-metastatic effects of periostin may also play a role in promoting intrapulmonary metastasis of lung cancer and this mechanism warrants investigation.

##### Thrombospondins

The glycoproteins of the thrombospondin family, primarily thrombospondin-1 and thrombospondin-2, are produced by immune, vascular and stromal cells within the lung and are known to inhibit the initiation of primary tumors and the outgrowth of secondary tumors in the lung (33, 91). One of the major mechanisms by which thrombospondin-1 regulates tumor outgrowth and dormancy is by interacting with the latency associated peptide in complex with TGFβ to release TGFβ into the local environment (92) and thereby stimulate TGFβ signaling (see Tenascin-C above). While this has been demonstrated *in vitro*, it is not clear if thrombospondin-TGFβ interactions regulate dormancy in the lung and at secondary lung cancer sites *in vivo*.

Studies in breast cancer models of lung metastasis have identified that cancer cells with high AXL expression also highly express thrombospondin-2 and this drives TGFβ1-dependent lung colonization (93). Upon colonization, interaction with fibroblasts in the lung triggers a phenotypic switch in the disseminated tumor cells to a proliferative, epithelial phenotype with activation of BMP-dependent SMAD 1-5 signaling, and downregulation of TGFβ-dependent SMAD 2/3 signaling to promote tumor outgrowth (93) (Figure 1D). In contrast, thrombospondin-2 expression by lung cancer cells promotes lung cancer bone metastasis through osteoclastogenesis (94) which may explain the association of this isoform with recurrence in adenocarcinoma (95). These data point to a role for thrombospondin-2 in promoting cancer cell proliferation in metastatic foci within the lung, although it is not clear what role this protein plays in primary tumor onset, progression, and evolution.

The role of thrombospondin-1 in lung tumors, however, remains contentious. Serum and intratumoral levels show conflicting associations with patient outcome (95–97), which may reflect differences in the release of thrombospondin-1 from the tumor microenvironment into the systemic circulation, or its specific localized effects within the tumor environment. This discrepancy may also be partly explained by differential effects of thrombospondin-1 on the initiation compared with the progression of lung tumors. Unlike thrombospondin-2, thrombospondin-1 acts as a tumor suppressor in pre-malignant Kras-mutant lung carcinoma, by inducing p53-dependent epithelial cell senescence (98). It is not known if this role for thrombospondin-1 is abrogated in the development of p53-mutant lung tumors, which lack functional DNA damage checkpoints. This may, however, point to a larger genetic dependency for the role of thrombospondin-1 in tumor progression. High MYC expression in breast and prostate cancer cell lines, which mimics MYC amplification seen in a significant proportion of NSCLC tumors, leads to the transcriptional upregulation of prosaponin (99). Prosaponin induces thrombospondin-1 *via* p53 activation in fibroblasts within the tumor environment, thereby increasing thrombospondin-1 levels in the local tumor microenvironment. The context dependency of thrombospondin-1’s involvement in lung cancer initiation, primary tumor progression and the establishment of secondary tumors remain to be clarified.

##### Osteopontin

Osteopontin is a secreted matrix–associated phosphoglycoprotein that is activated by MMP3/7 or thrombin protease (100, 101). High expression of osteopontin is associated with poor survival and tumor aggressiveness in NSCLC (102, 103). Osteopontin interacts directly with α4- and α9β1 integrins to promote tumor cell invasion (104), with the alternatively spliced C-terminally truncated form of the protein being particularly pro-invasive (105). Follistatin-like protein 1, which regulates alveolar maturation in the developing lung by inhibiting BMP4/SMAD1/3/5 signaling (106) and inhibits emphysema development in response to injury (107) also binds directly to pro-osteopontin to prevent its proteolytic activation and thus inhibit lung cancer metastasis. This likely underpins why the expression of follistatin-like 1 protein is downregulated in NSCLC and low expression of this protein is associated with poor survival (101).

#### Proteoglycans and Glycosaminoglycans

Proteoglycans have a central protein core from which covalently attached linear repeating glycosaminoglycan chains or sulfated polysaccharides extend outward. Proteoglycans are considered a subclass of glycoproteins that specifically contain unbranched and repeating O-linked carbohydrate molecules called glycosaminoglycans. The major glycosaminoglycan classes are hyaluronan, chondroitin, dermatan, heparin and keratan. Proteoglycans are commonly secreted by cells and are enriched in the basement membrane where they act as nucleating components for the fibrillogenesis of collagens and the assembly of elastin fibers (108, 109).

##### Versican

Versican is a chondroitin sulfate proteoglycan that regulates the perialveolar tissue volume and contributes to alveolar maturation during lung development (110). Although the mechanisms governing versican’s role in lung development remain unclear, its interaction with CD44 and β1-integrin is likely to play an important role in these processes. In lung cancer, versican is highly expressed in NSCLC and its expression is tightly correlated with hyaluronan (111). The particular association of versican with patient outcome in adenocarcinoma, but not squamous NSCLC (111) also suggests that its effects may be specific to the progression of the adenocarcinoma histological subtype.

Versican has been shown to be produced by lung cancer cells and activate alveolar macrophages *via* TLR2, TLR6 and CD14 signaling to induce TNFα and IL6 production that subsequently supports lung colonization (112). The association of versican with proliferative signaling in breast, brain, prostate and melanoma cancer cells (113), suggests it may also play a role in regulating the growth of both primary tumors and metastatic foci alike.

##### Hyaluronan

Hyaluronan is an unbranched heteropolysaccharide glycosaminoglycan that does not form proteoglycans but non-covalently interacts with proteoglycans and other ECM components. It is present within the basement membrane of bronchial and bronchiolar epithelium, within the perivascular region of large blood vessels, and on the cell surface of alveolar macrophages in the healthy lung. CD44, the main cellular receptor for hyaluronan (Figures 1B,C), is localized to the basolateral surface of the bronchial epithelia, enabling these cells to bind to hyaluronan in the basement membrane. CD44 expression is increasingly associated with the stemness characteristics of cancer cells, although the presence of cancer stem cells in NSCLC subtypes remains equivocal. At present it also remains unclear whether ECM-CD44 engagement drives stemness phenotypes, or whether the engagement of already upregulated CD44 ligands with ECM components such as hyaluronan preferentially supports cancer cells with stem-like phenotypes. In the normal lung, hyaluronan is expressed by multiple cell types in response to injury, where it is involved in the fibrosis and wound repair response and modulates lung inflammation (114). However, in lung cancer, hyaluronan has been shown to promote the outgrowth of NSCLC tumors (39). During tumor progression, high p38 expression in cancer-associated fibroblasts also induces hyaluronan deposition into the lung tumor environment that subsequently promotes the proliferation and outgrowth of NSCLC (115). Hyaluronan may also promote the colonization and outgrowth of distant or intrapulmonary metastases, since high hyaluronan levels are associated with increased recurrence rates in lung adenocarcinoma (116). Similarly, high expression of the hyaluronan receptor CD44 on NSCLC cells is associated with squamous histology, as well as increased metastasis and poor survival in adenocarcinoma patients (117). In response to hyaluronan, CD44 receptor engagement activates multiple downstream signaling pathways including activating TGFβ signaling as well as Nanog-Stat2, Oct4-Sox2-Nanog and c-Src-kinase signaling that culminate in STAT3 or CBP/p300 mediated transcriptional reprogramming (including inducing MMP, cyclin-D1, pro-survival, and EMT gene expression) as well as actin cytoskeletal remodeling (Figures 1C–E). Together these collectively promote the self-renewal capacity of lung cancer cells, support the survival of cells at the primary tumor site and prime them for metastatic dissemination and colonization (118, 119).

Age-related loss of the hyaluronan and proteoglycan link protein 1 (HAPLN1), which directly binds hyaluronan (120), has also been associated with increased lymph node permeability and increased lymphatic metastasis in melanoma (121, 122). While it is not clear if this mechanism also operates in lung cancer, it reflects broader changes in extracellular matrix secretion by pulmonary fibroblasts seen during aging and paralleled in chronic lung diseases (123), that may explain observed increases in metastatic propensity associated with age in this NSCLC.

## Matrisome-Associated Factors in Lung Dormancy

Structural matrisomal proteins in the lung extracellular matrix directly interact with soluble growth factors and ECM degrading enzymes (124). Secreted factors specific to the lung are likely to contribute to the lung tropic characteristics of dormancy and outgrowth.

Many proteins that associate with the core matrisome regulate dormancy and outgrowth in a tissue- and context-specific manner. The most prominent of these is TGFβ, which is physically tethered to the matrix and is released upon matrix remodeling. The lung has a uniquely high expression of the TGFβ superfamily member BMP4, which promotes the dormancy of disseminated tumor cells within this tissue (125). In breast cancer models, disseminated cancer cells can overcome the suppressive microenvironment of the lung by expressing Coco, which directly binds to BMP4, preventing the activation of TGF receptors and triggering the re-awakening of dormant cells (125) (Figure 1B). Importantly, this mechanism appears to be specific to lung tissue and may explain the lung tropic behavior of non-pulmonary primary tumors. Low endogenous levels of another ligand of the TGFβ superfamily, TGFβ2, in the lungs has also been proposed as a mechanism supporting the outgrowth of disseminated tumor cells and may explain the short latency of metastatic foci in the lungs compared with other organs (126). However, it is not clear if these same mechanisms contribute to the dynamics of primary lung tumors or their intrapulmonary metastases.

The canonical ligands regulating stemness also interact with the extracellular matrix and modulate the metastatic capacity and dormancy phenotypes of cancer cells at primary and secondary sites. Both lung cancer cells and disseminated cancer cells that colonize the lung are enriched for stemness characteristics (127). This stem-cell like population of lung and breast cancer cells are more responsive to collagen remodeling and integrin-mediated reawakening mechanisms (28, 128). Wnt and Notch signaling are fundamental pathways that regulate these stemness characteristics of NSCLC cells and support the survival of disseminated cancer cells in the lung (129–131) (Figure 1E). Lung and breast cancer cells with high expression of the Wnt inhibitor DKK1 and the stemness factor SOX2 survive in dormant colonies within the lung, brain and kidneys (28). In this context, high DKK1 and SOX2 expression induces a slow proliferative state and self-renewal phenotype that enables these disseminated cells to persist. Furthermore, DKK1-driven evasion from Natural Killer (NK) cell–mediated clearance by downregulation of NK cell activators supports the maintenance of these dormant colonies (28). High expression of DKK1 also mediates the outgrowth of bone-colonizing lung adenocarcinoma cells by inhibiting osteoblast differentiation in the pre-metastatic niche as well as the induction of the Wnt signaling transcription factor LEF1 and embryonic development gene HOXB9 (132, 133). The interaction of extracellular matrix components with Wnt ligands regulates their availability to induce the proliferation and differentiation of multiple pulmonary cell types as well as lung cancer cells (133).

The association of these signaling effectors with the core matrisome in the lung creates a dynamic functional network that directs the behavior of primary and disseminated cancer cells alike. An improved understanding of how the dynamics of these protein-protein interactions influence cancer cell dormancy and dissemination will generate a more complete picture of the role of the extracellular matrix as a functional, as well as a structural component of the tumor microenvironment. The dynamics of extracellular matrix synthesis and turnover, which regulates the release and sequestration of these matrisome-associated factors, is therefore a significant consideration in the functional dynamics of the tumor microenvironment.

## The Dynamic Matrix and Its ECM Remodeling Factors

The extracellular matrix is a dynamic component of the tumor microenvironment that regulates the capacity of tumor cells to migrate away from the primary tumor and colonize local or distant sites to form overt metastases. The structure and composition of the extracellular matrix is remodeled by multiple enzymes, such as the lysyl oxidases, MMPs and ADAMTS families that mediate both the crosslinking and degradation of ECM components. The degradation products of these remodeling processes are far from passive byproducts. Many of these products of catabolic ECM remodeling have individual signaling roles that mediate crosstalk between cell types within the tissue environment or are released systemically to influence the function of multiple organ systems. Therefore the dynamic remodeling by these enzymes significantly contributes not only to the ECM composition within the local tissue environment but also to the functional responses of cells within both the local and systemic environments.

### ECM Cross-Linking and Stabilization

Collagen architecture is largely built up by the crosslinking of collagen fibrils to form highly bundled collagen fibers. Collagen crosslinking is initiated by lysyl hydroxylases, which catalyze the intracellular hydroxylation of lysine residues. Lysyl hydroxylase 2 is highly expressed in adenocarcinoma, where it promotes NSCLC metastasis by increasing tumor stiffness to activate cancer cell invasion, PI3K signaling and modulates collagen remodeling (134–136). These studies indicate that although lysyl hydroxylases typically operate on intracellular collagen at the endoplasmic reticulum, lysyl hydroxylase 2 is also secreted into the extracellular compartment of the tumor microenvironment where it modifies collagen fibers (136). It is not yet clear if the intracellular or extracellular activity of lysyl hydroxylase-2 more significantly contributes to its pro-metastatic effect.

Enzymes of the lysyl oxidase family catalyze the oxidative deamination of hydroxy-lysine and lysine to reactive aldehydes once the collagen has been secreted into the extracellular compartment. These reactive aldehydes can then spontaneously crosslink neighboring fibers. This lysyl oxidase-mediated collagen cross-linking is essential to the formation of mature collagen fibrils and fibers that underpin the structural integrity of the collagen matrix. This highly crosslinked collagen is also more resistant to MMP–mediated degradation (137) and the crosslink density is known to determine the stiffness of the lung tissue (138).

In non-pulmonary cancers, the lysyl oxidase family profoundly promotes the dissemination of cancer cells from the primary site as well as the colonization of and outgrowth at distant sites, including the lung, through direct effects on collagen architecture and tissue biomechanics (41, 139–144), and through the recruitment of myeloid cells to the premetastatic niche (68). The secretion of lysyl oxidase (LOX) by tumor cells in response to intratumoral hypoxia, or as a wound healing response to surgery, can increase the systemic LOX activity, thereby catalyzing collagen crosslinking at distant sites, including within the lung (68, 145). This systemically elevated LOX activity can therefore promote metastatic colonization and outgrowth at distant sites. In NSCLC, the expression of LOX and lysyl oxidase-like 2 (LOXL2) are upregulated and increase the metastatic potential of lung tumor cells (144). These enzymes are insufficient to drive cancer cell invasion alone (144) and instead appear to operate in concert with mesenchymal phenotypic programs to facilitate dissemination.

The importance of lysyl oxidases in lung cancer dynamics is dependent on the histological subtype and genomic profile of the tumor. In particular, loss of LKB1, which occurs in 34% of adenocarcinoma and 19% of squamous carcinomas (146), induces mTOR-HIF1α signaling to induce lysyl oxidase expression within the tumor microenvironment. Lysyl oxidase-mediated collagen crosslinking then increases the stiffness of the tumor tissue and activates pro-proliferative β1-integrin signaling (142). Interestingly, increased LOX activity in this context is also associated with increased transdifferentiation of adenocarcinoma cells toward a squamous phenotype (142), suggesting that remodeling of the local ECM and increases in tissue stiffness may contribute to the development of the squamous histological subtype of NSCLC.

### ECM Turnover

Proteases play an important role in the degradation and turnover of all matrix components in the lung and at distant sites. In the lung, neutrophil-derived MMP9, cathepsin G and elastase cleave laminin-111 (61) and thrombospondin-1 (147) to promote the outgrowth of cancer cells within the lung. High MMP2 and MMP9 expression are also associated with increased intrapulmonary metastatic potential of lung adenocarcinoma (148). In addition, high MMP13 expression drives the colonization of the brain by lung adenocarcinoma cells, but was not found to functionally influence the growth of these micrometastases after their colonization (8).

Proteases within the lung environment also cleave ECM components to release growth factor ligands that regulate pro-proliferative intracellular signaling events. For example, BMP1 cleaves latency associated binding peptides thereby releasing TGFβ into the microenvironment to promote dormancy (149). Conversely, MMP14 expressed by NSCLC cancer cells and myeloid cells in the tumor microenvironment digests the heparin-binding EGF-like growth factor (HB-EGF) to generate both soluble and membrane-bound heparin-independent growth factors that can activate EGFR signaling (150, 151). EGF-like signaling ligands are also released by MMP14-mediated degradation of the laminin 5 γ2 chain to generate EGF-like fragments that drive EGFR signaling toward increased NSCLC tumor growth (150, 151). Similar MMP14-mediated remodeling operates in the wound healing response of the normal lung to regenerate alveoli (150, 151), supporting the notion that aberrant wound healing responses significantly contribute to disease progression in NSCLC. Amplification of another member of the metalloproteinase family, MMP13, promotes brain metastases specifically in Kras-mutant lung adenocarcinoma (8). Although the precise mechanism underlying this association has not yet been dissected, this does suggest that the role of MMPs in lung tumor dissemination is likely to depend on the genetic profile of the tumor.

Proteases that directly modify core matrisomal proteins also activate other ECM remodeling enzymes. For example, BMP1 which activates fibrillar pro-collagens by removing their N and C-termini, also cleaves the pro-LOX protein to release the mature active enzyme (45). This pleiotropic activity synchronizes extracellular matrix secretion and deposition with its stabilization and remodeling within the microenvironment. Furthermore, the pleiotropic activity of BMP1 and other proteases in releasing growth factor ligands that are either tethered to the extracellular matrix (e.g., TGFβ), or as motifs sequestered within intact protein targets of these proteases (e.g., EGF-like domains), also orchestrates both ECM and growth factor signaling (152), which converge on the same intracellular signaling pathways (Figure 1D). This synchronization of extracellular events and the symmetry of intracellular signaling means that extracellular matrix remodeling can generate profound changes in cancer cell behavior to collectively regulate tumor dynamics by multiple mechanisms.

## ECM Biomechanics in Lung Cancer Progression and Dormancy

The network behavior of matrisomal proteins determines the biomechanical properties of lung tissue. Elevated fibrillar collagen content and higher levels of crosslinking between collagen fibrils in NSCLC tumors increases the mechanical stiffness of the tissue (138). Mechanosensitive receptors then activate intracellular signaling networks that promote the dissemination of cells away from the primary tumor and support the establishment of intrapulmonary and distant metastases.

The increased stiffness of the lung tumor environment, driven by many of the processes described above, is sensed by lung fibroblasts through a number of mechanisms, including β1-integrin engagement. This mechanosignaling then directs their transition to a myofibroblast-like phenotype that secretes high levels of matrisomal proteins as well as ECM remodeling enzymes (153). This mechanoresponsive signaling creates a self-reinforcing amplification loop that further increases the ECM stiffness in the tumor microenvironment to promote the growth of primary NSCLC tumors (154–156) (Figure 1E). In *in vitro* models, this mechanoresponsive fibroblast activation can persist for several weeks once the matrix stiffness is reduced, suggesting that transient stiffening of lung tissue in response to injury may also induce long lasting effects on cellular behavior that can potentiate tumorigenic processes (157). In experimental models of idiopathic pulmonary fibrosis, stiff matrices signal through FAK and Rho kinase in lung fibroblasts to regulate multiple downstream pathways including YAP/TAZ, which converge on apoptotic mediators to inhibit fibroblast apoptosis, as well as further accelerating pro-fibrotic remodeling (158). Overlapping mechanisms are seen in NSCLC, where stiff matrices also drive FAK activation and β-catenin accumulation that increase the responsiveness of intracellular Wnt signaling and drive self-renewal programs in NSCLC cells (159). These effects are also seen in breast cancer models where stiff matrices promote focal adhesion assembly and enhance cytoskeletal tension to increase the activation of ERK and PI3K in response to growth factor ligands in tumor cells (160–162). In pancreatic ductal adenocarcinoma cells, oncogenic Ras activity can further amplify these responses by stimulating ROCK activity to subsequently stiffen the extracellular matrix and drive tumor growth (163), although this is yet to be demonstrated in non-small cell lung cancer where activating Ras mutations are common.

Lung tissue is under constant mechanical stress due to strains induced during normal breathing. On a micro scale, cellular contraction pulls on the extracellular matrix components to also generate strain within the lung. The tensile and compressive strain within the lung extracellular matrix regulates the sequestration and release of matrix-associated factors. For example, TGFβ is tethered to the extracellular matrix by latent TGFβ binding proteins, which interact with fibronectin, fibrillins, fibulins, proteoglycans, and other fibrillar ECM components, as well as the latency associated peptide (LAP), which binds to integrin receptors (73, 81, 82). Mechanical tension induced by cellular contraction, including by highly contractile pulmonary myofibroblasts (164), causes a conformational change in the TGFβ-LAP complex that releases the TGFβ ligand and enables it to activate the TGFβ receptor (73, 81). While this operates at a low level to drive localized lung regeneration in the wound healing response, the severe impairment of lung regeneration in idiopathic pulmonary fibrosis causes extensive alveolar loss that significantly elevates the tensile forces that type II pneumocytes are exposed to (165). This increases the release of TGFβ into the alveolar environment to activate pro-fibrotic TGFβ signaling in lung fibroblasts (165). Because alveolar loss occurs in the periphery, this generates progressive fibrosis from the distal parenchyma toward the central airways, that is a feature of idiopathic pulmonary fibrosis progression. This newly described mechanism raises the possibility that altered force distribution throughout the lung as a result of perturbed ECM remodeling in lung tumors or in chronic lung diseases, including COPD, may also induce TGFβ signaling to support tumor dormancy or, conversely, a pro-fibrotic environment that drives cancer cell proliferation. This mechanism may partly explain why IPF patients are at increased risk of developing lung tumors and why these tumors develop close to the fibrotic regions in IPF lungs (24, 166, 167).

Integrin signaling is responsive to mechanical strain within lung tissue, which occurs during normal breathing and is perturbed by ECM remodeling. In addition to having different affinities for different ECM components, integrin receptor subtypes display different temporal kinetics in response to mechanical force, allowing them to activate different mechanotransduction pathways in response to specific changes in lung biomechanics (168). For example, cyclic strain, which mimics breathing biomechanics, induces gefitinib resistance *via* β1-integrin signaling in EGFR mutant lung adenocarcinoma (169). Integrin clustering also mediates downstream signaling diversity, while stress-induced conformational changes in focal adhesion proteins, such as talin, can fine-tune the response of cells to the magnitude of local forces (170). Moreover, the biomechanics of the tumor microenvironment feed back onto the architecture of the extracellular matrix itself. For example, fibronectin assembly and collagen fibrillogenesis are both regulated by tension applied to fibronectin (65, 109, 171). In this way the lung biomechanics create positive and negative amplification loops that can accelerate the outgrowth of primary and disseminated tumor cells or drive tumor cells into sustained dormancy.

## Stromal Cells and Their Roles in Dormancy and Outgrowth

### Fibroblasts

**FIGURE 2:**
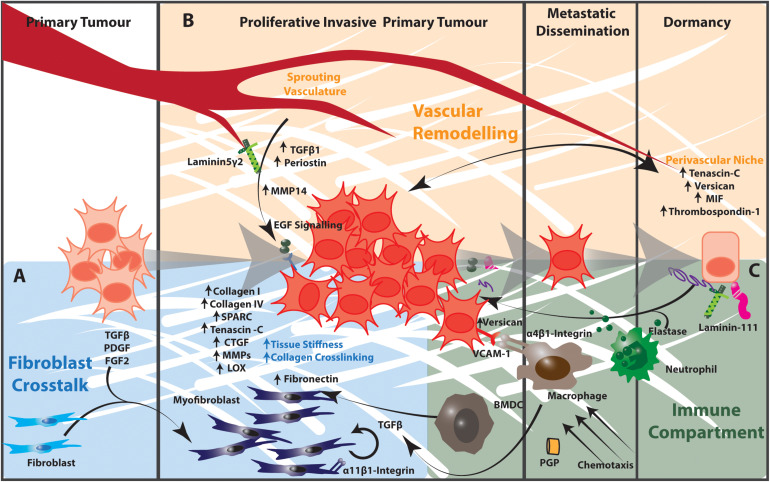
The tumor microenvironment contributes to matrix remodeling that regulates tumor cell dormancy and outgrowth in NSCLC. **(A)** Cancer cells release TGFβ, PDGF, and FGF2, which activate fibroblasts to a myofibroblast state. Activated myofibroblasts in the tumor microenvironment produce increased amounts of collagen I, collagen IV, Tenascin C, Thrombospondin-2 as well as remodeling enzymes and signaling effectors such as CTGF and MMPs. The resulting increased matrix stiffness further activates fibroblasts in a TGFβ-dependent manner. Stromal cells expressing α11β1-integrin engage with this extracellular matrix to promote the proliferation, migration, invasion and survival of cancer cells in the tumor microenvironment. **(B)** The extracellular matrix components of the perivascular niche support dormancy in cancer cells. The sprouting vasculature produces TGFβ and periostin that promote cancer cell proliferation and metastatic potential. Endothelial cells of the sprouting vasculature also produce MMP14, which cleaves laminin 5 γ2 to release EGF-like fragments that activate EGF signaling in cancer cells. **(C)** Activated infiltrating macrophages secrete TGFβ into the tumor microenvironment to activate fibroblasts and TGFβ-dependent signaling in cancer cells. α4β1-Integrin-expressing macrophages engage with VCAM-1 expressing cancer cells to induce pro-survival Akt-PI3K signaling. Proteases produced by neutrophils and other immune cells cleave ECM components including elastin and thrombospondin-1. These cleavage products awaken dormant cancer cells and act as chemotactic agents to recruit immune cells into the tumor microenvironment. Collectively the stromal and immunological components of the tumor microenvironment regulate cancer cell dormancy and outgrowth in the lung. BMDC, bone marrow-derived progenitor cell.

As the major producer of extracellular matrix components, lung fibroblasts play an important role in regulating the lung ECM in both health and disease. The lung fibroblast population is highly heterogeneous and the aberrant expansion of specific fibroblast phenotypes contributes to ECM remodeling in lung tumors. In addition to the effects of increased matrix stiffness discussed above, growth factors such as TGFβ, PDGF and FGF2 secreted by cancer cells and tumor-infiltrating immune cells recruit and activate fibroblasts to a myofibroblast-like state (Figure 2). This state is characterized by transcriptional and signaling programs that promote fibroblast proliferation, inhibit apoptosis and drive ECM remodeling leading to increased mechanical stiffness. This increased matrix stiffness within the tumor microenvironment creates a TGFβ-dependent amplification loop that further increases both ECM stiffness and fibroblast activation (172–174). These activated fibroblasts also secrete large amounts of collagen I, collagen IV, extra domain A-fibronectin, heparin sulfate proteoglycans, secreted protein acidic and rich in cysteine (SPARC), tenascin–C, thrombospondin-2, connective tissue growth factor, MMPs and plasminogen activators. These each contribute to the significant remodeling of the matrix in the primary lung tumor microenvironment and subsequently alter the behavior of tumor and neighboring untransformed epithelia (93, 172, 174–177).

Multiple transcriptional programs have been identified that distinguish cancer-associated fibroblasts from healthy lung fibroblasts in NSCLC (40, 174, 178). These transcriptional signatures are associated with poor prognosis in NSCLC and center on genes that modulate cell-ECM interactions (40, 174, 178). In addition, increased matrix stiffness due to higher levels of collagen crosslinking within the tumor activates stromal α11β1-integrin signaling to promote the proliferation and increase the metastatic potential of NSCLC cancer cells (154). Emerging evidence indicates that the somatic mutational profile of pancreatic cancer cells can contribute to these phenotypic perturbations by re-educating their surrounding stroma and tissue architecture through paracrine mechanisms to further promote tumor aggressiveness (179). It remains to be determined if particular mutational profiles in non-small cell lung cancer cells drive the specific reprogramming of nearby resident lung fibroblasts in a similar way to indirectly support tumorigenesis.

The metabolic activity of cancer-associated fibroblasts has also been recognized as a means of regulating ECM-dependent dormancy in addition to more established bioenergetic co-dependencies between these cell types (180). Cancer-associated fibroblasts have increased glycolytic and autophagic activity compared with normal lung fibroblasts, and have been shown to promote rapid cancer cell proliferation as well as maintain cancer-associated fibroblast survival (27, 180). Since collagen turnover is partially regulated by autophagic proteins (181), the increased autophagic activity of cancer-associated fibroblasts may support more rapid collagen remodeling and an increasingly dynamic extracellular matrix framework. Furthermore, as autophagy activation integrates quiescence and survival signals (27), it is plausible that collagen fibrillogenesis may reciprocally influence autophagy activity to regulate cell survival and dormancy in the lung.

Finally, the long range stiffness gradients generated by fibroblast-mediated ECM remodeling (182) may contribute to field of cancerization effects within the lung, which may promote intrapulmonary metastasis. Crosstalk between epithelial cells and fibroblast subtypes within different lung compartments would generate location-specific regulatory loops that specify the dormancy and outgrowth behavior of tumor cells in a spatial manner. How these processes might contribute to the heterogeneity within and between tumors has not been elucidated. An improved understanding of spatial cues within and between lung compartments may reveal cell extrinsic mechanisms that dictate the emergence of different primary lung cancer subtypes in specific anatomical locations, the character of heterogeneous regions within individual primary tumor masses as well as the location of metastatic foci within the lung.

### Immune Cells

Inflammation plays a major role in lung tumorigenesis and etiology (183) and emerging evidence points to extracellular matrix remodeling as both a consequence and instigator of inflammatory processes within the lung. While a comprehensive understanding of the immunological landscape of the lung during the metastatic dissemination, dormancy and reawakening is lacking, emerging evidence is revealing that immune cells interact with the extracellular matrix to contribute to these processes. The different immunological landscapes associated with specific oncogenic driver mutations in NSCLC (184) may also contribute to the heterogeneity in extracellular matrix remodeling during tumor progression.

Immune cells can re-educate resident fibroblasts in and around tumors, and likely play an important role in establishing a permissive environment that supports lung colonization and outgrowth. In addition, immune cells are capable of synthesizing extracellular matrix proteins, although they not a major source of extracellular matrix components within tissues. For example, myeloid cells produce versican (185) while activated and infiltrating macrophages secrete TGFβ to induce the pro-fibrotic transformation of resident fibroblasts. Immune cells also remodel extracellular matrix proteins to regulate cancer cell dormancy and the local immune landscape, while the proteolytic products of these reactions also recruit and activate other immune cells. For example, as discussed above, neutrophil–derived proteases such as elastase cleave laminin-111 and thrombospondin-1 to awaken dormant breast cancer cells within the lung (61, 147), and the cleavage products of elastin are highly chemotactic for monocytes (Figure 2) (186). Furthermore, the degradation product of type I collagen, Proline-Glycine-Proline tripeptide (PGP), mimics CXC chemokines such as IL-8 and interacts with the CXCR1 and CXCR2 receptors to attract neutrophils to the ECM remodeling site (187). This further amplifies extracellular matrix remodeling within the tumor microenvironment and regulates the proliferative behavior of tumor cells in this environment.

These tumor infiltrating immune cells also directly interact with disseminated tumor cells in the lungs to regulate their proliferative state through ECM responsive integrin and TGFβ signaling. Activated α4β1 integrin-expressing macrophages associate with VCAM-1 positive cancer cells in the lung to induce pro-survival Akt-PI3K signaling in tumor cells (188). Similarly, bone-marrow derived progenitors recruited to the premetastatic lung induce the mesenchymal to epithelial transition of tumor cells through downregulation of SMAD2 signaling in the canonical TGFβ pathway, and a switch to macrometastatic growth (185). Characterization of the NSCLC immune landscape will further define how complex interactions between these immune cells, cancer cells and stromal cells within the tumor microenvironment modulate the extracellular matrix, and subsequently the dynamics, of primary and secondary NSCLC tumors.

### Vascular Architecture and Dormancy

The dynamics of the pulmonary capillary also regulates tumor cell dormancy and outgrowth, although primarily in the context of metastasis to the lung from non-lung primary tumors. Once primary tumor cells have disseminated from the primary tumor site, contact with the epithelial basement membrane is replaced by binding to the endothelial basement membrane that surrounds capillaries. In metastatic models of primary lung tumors, the metastatic colonization of distant sites involves extravasation of lung tumor cells at vascular branch points and the outgrowth of tumors at these perivascular locations (189). While the physical size of the capillary bed may play a role in physically trapping disseminated tumor cells in the pulmonary capillary bed or at vascular branches in distant organs, this perivascular niche that surrounds the vasculature also presents specific cues that regulate the proliferation of lung epithelia and disseminated tumor cells alike (151). The destabilization of pulmonary vasculature occurs early in NSCLC as well as in breast cancer metastasis to the lung (190–192), supporting the notion that remodeling of the perivascular niche significantly contributes to tumor progression.

In breast cancer metastasis, endothelial tip cells within the perivascular niche deposit increased levels of periostin, tenascin-C, versican, S100 proteins, TGFβ and MIF, which act to maintain cancer cell dormancy (Figure 2) (193). Non-sprouting endothelial cells also produce thrombospondin-1 which associates with the mature microvascular basement membrane to sustain quiescence (89). Conversely, the production of TGFβ1 and periostin by tip cells of the sprouting neovasculature can promote tumor outgrowth (89). In this way cancer cells become dormant in the presence of dormant microvasculature, and become reactivated during angiogenic sprouting through interactions with the extracellular matrix of the perivascular niche. Furthermore, VEGF and FGF signaling in pulmonary endothelial cells induces MMP14 expression, which releases EGF-like ligands from the laminin 5 γ2 chain and drives pro-proliferative EGF signaling in epithelial cells (151). Together these mechanisms link the remodeling of the extracellular matrix within the perivascular niche to the proliferative behavior of disseminated tumor cells.

## Exploiting the Tumor ECM to Improve Clinical Practice

### Using the ECM to Inform Clinical Decision Making

The extracellular matrix features that characterize NSCLC patients at high risk of poor outcome outlined above have the potential to be utilized as clinical decision-making tools in determining the most effective therapy for a patient’s condition. In NSCLC, where there are currently no clinicopathological features identifying early-stage patients at high risk of disease recurrence following surgery, there is a clear need to identify the distinguishing characteristics of this population. Transcriptional signatures associating matrisomal gene expression with lung cancer recurrence holds promise that such approaches may robustly identify early stage patients at high risk. This will become more important as early stage diagnoses continue to increase (11). Improvements in treating NSCLC will require an understanding of the burden of disseminated tumor cell dormancy at diagnosis, as well as the relative threat posed by the likely presence of metastatic niches and dormant disseminated tumor cells in terms of their ability to grow into macrometastases, or alternatively remain dormant for many decades.

The substantial progress in understanding the extracellular matrix dynamics occurring during primary tumor development and metastatic colonization of many tumor types to date provides hope that this knowledge may improve clinical practice in the future. Although, our understanding of these processes in lung cancer is lagging behind that of other common cancers, such as breast cancer. Of critical importance in realizing this goal will be developing biomarkers of these processes that perform with satisfactory sensitivity and specificity. Moreover, it remains a challenge to develop tests that are capable of extending beyond robust performance on population level data to predict risk for an individual patient. Such readouts must also be readily appropriated to existing clinical management pipelines.

This understanding could also establish a path to develop therapies targeting tumor cell dormancy in NSCLC. Dormant cells largely escape immune surveillance and are resistant to conventional and targeted chemotherapies (194). Therefore, strategies to treat metastasis by targeting tumor dormancy follow two main approaches (33): (1) trap disseminated tumor cells in a dormant state in the long term or (2) reawaken dormant cells into a proliferating state and eradicate them using standard of care treatments. Distinguishing between patients where dormant cells are at high risk of becoming reactivated, for example by extracellular matrix remodeling in response to injury or environmental exposure, compared with those that may remain dormant indefinitely, is key to effectively managing cancer as a chronic and ultimately curable disease.

A deeper understanding of the role of the tumor microenvironment will also assist in determining which existing therapies are the most appropriate for the patient. It will also be important to determine whether surgical resection in early stage cancers is likely to increase the risk of activating already disseminated tumor cells and therefore worsening the patient prognosis (145). Similarly, chemotherapy- or radiotherapy-induced lung fibrosis may also accelerate the awakening of otherwise dormant cancer cells in the lung (195). Some conventional chemotherapy agents that induce DNA damage may also activate quiescent cells to re-enter the cell cycle (33) and may be preferred in patients suspected to have a high burden of dormant disseminated tumor cells. Conversely, existing therapies that are effective at inducing dormancy in disseminated cells may be used as maintenance therapies to keep these cells in a dormant state (34, 196, 197). For example, the EGFR targeted therapy erlotinib in combination with a BH3 mimetic induced prolonged quiescence in preclinical models of NSCLC xenografts (198), suggesting that therapeutic approaches that achieve disease control in the long term may require combination therapies that simultaneously inhibit mitogenic and apoptotic signaling pathways. Whether these approaches can be applied in the stromal targeting context remains to be seen.

However, consideration must be given to the impact of such therapies on stromal cells within the tumor microenvironment. For example, cisplatin treatment induces AXL and GAS6 expression by cancer associated fibroblasts to promote the migration of AXL-expressing lung cancer cells (199). An increasing number of clinical trials testing the ability of conventional therapies to target disseminated tumor cells as primary endpoints of their clinical trials (200) coupled with technological advancements to detect disseminated tumor cells, will be critical to facilitate the testing of more ECM-centric targeted therapies that reawaken or eradicate dormant tumor cells.

### Novel Therapeutic Targeting of the ECM

Recent advances in our understanding of how the matrix is impacting tumor cell dormancy has also led to the development of novel therapeutic strategies aimed at maintaining dormancy or eradicating dormant cells.

The recognition of common fibrotic mechanisms in idiopathic pulmonary fibrosis and lung cancer has led to interest in repurposing anti-fibrotic IPF therapies to the treatment of lung cancer. Many anti-fibrotic therapies have met limited success in clinical trials for their ability to inhibit metastatic colonization and promote dormancy maintenance. However, the VEGF, PDGF and FGF inhibitor Nintedanib, which is used in the treatment of idiopathic pulmonary fibrosis, has been approved in some jurisdictions as a second line therapy in combination with conventional chemotherapy (e.g., pemetrexed) in NSCLC and renal cell carcinoma (201). By blocking the activation of fibroblasts to myofibroblasts, it significantly reduces the degree of ECM remodeling within tumors. Pirfenidone, the other approved anti-fibrotic IPF therapy, is still being tested for its efficacy as a NSCLC treatment. Other IPF treatments targeting galectin-3 (TD139), TGFβ (GC1008), αvβ6 integrin signaling (BG0011, GSK3008348) and inflammatory mediators (QAX576, Carlumab) are currently under clinical development for IPF but may yet find utility as NSCLC therapies (24). The lack of treatments targeting mechanisms underlying COPD precludes repurposing of these agents into the oncology space, although it is hoped that emerging treatments for COPD may also prove efficacious in treating lung cancer patients.

Additionally, LOX-neutralizing antibodies that block collagen I deposition and cross-linking have shown utility in preclinical studies targeting metastasis of non-pulmonary tumors to the lung (68, 143). Preclinical development of LOX inhibitors for non-pulmonary tumors are ongoing, although their efficacy in NSCLC remains untested. Antibody targeting of LOXL2, which is the other member of the lysyl oxidase family that is highly expressed in NSCLC, have yielded disappointing results in clinical trials in pancreatic and metastatic colorectal cancer (202, 203) but, like LOX inhibitors, may yet prove effective in NSCLC treatment.

Directly targeting the extracellular matrix components themselves has also shown promise. Antibodies against the pro-proliferative proteolytic fragment of laminin-111 suppressed the outgrowth of breast cancer metastases in the lungs in a preclinical model (61), although its efficacy in primary lung tumors has not yet been tested. Glycoproteins or short peptides derived from quiescence-inducing proteins such as the thrombospondin-1-inducing glycoprotein prosaposin have successfully induced systemic thrombospondin-1 to inhibit the metastatic outgrowth of prostate and breast cancer lung metastases (91, 99). However, due to the contentious role of thrombospondin proteins in NSCLC progression, it is not yet clear whether this approach will also be effective in inhibiting the growth of primary lung tumors or their intrapulmonary metastases.

Other experimental approaches are targeting key signaling processes downstream of matrix engagement. Suppression of MAPK signaling, or by targeting EGFR, MMP9, amphiregulin, FAK, SRC kinases, ROCK or PI3K, as well as by sustaining or inducing p38 and HOXD10 signaling have proven effective at inducing dormancy in breast and pancreatic cancer models (193, 204) and similar approaches may be effective in NSCLC. Targeting MLCK to inhibit actin remodeling that switches cells from dormancy to proliferation in response to the extracellular matrix is also being explored in breast cancer and osteosarcoma models that metastasize to the lung (49). α5β1- and α5β3- integrin targeting antibodies, cyclic peptides or peptidomimetics have also been trialed to suppress lung colonization and outgrowth in breast cancer metastasis but have yielded disappointing clinical trial results (53). Similarly, integrin-targeting antibodies are being developed as PET imaging tracers for the diagnosis of cancer (205).

Other approaches are using matrix component epitopes to target drugs directly to the tumor site. For example, the collagen-binding properties of lumican have been exploited to target collagen-rich melanoma tumor environments with interleukins (206) and may be applicable to the high collagen content of NSCLC tumors. Similarly, immune checkpoint therapies conjugated to a heparin binding domain peptide with a high affinity for glycoproteins and some collagen proteins have also shown promise in preclinical studies in melanoma and breast cancer models (207). Matrix-targeting nanobodies are also being developed as PET tracers which may be adapted to mediate ECM-targeted drug delivery (208). The diverse and complementary approaches being pursued to exploit the role of the extracellular matrix in tumor dormancy and outgrowth provide promise that effective therapies will be developed to manage NSCLC cancer as a chronic disease.

## Future Challenges

While tremendous progress has been made in understanding the complex interplay between cancer cells, the extracellular matrix and the surrounding stromal cells in regulating the dynamics of primary and secondary tumors in many cancer types, the interrelated functions of these components in lung cancer are still being revealed. The complex interplay of ECM components and cell types within the healthy lung, coupled with the highly heterogeneous landscape of lung tumors themselves make this particularly challenging. Defining the phenotypic plasticity of these cell types in healthy and diseased tissue will be fundamental to determining how crosstalk between these cell types orchestrates the extracellular matrix composition of primary and secondary lung tumors. This is particularly the case for fibroblasts and epithelial cells, where lineage-tracing experiments will be invaluable in revealing the extent to which different cell types assume the myofibroblast phenotype in the tumor microenvironment or pre-metastatic niche. Furthermore, as a load-bearing organ, consideration should be given to the mechanical cues that regulate cell behavior within these lung compartments.

The development of *in vitro* and *in vivo* models that more accurately recapitulate the dynamic ECM remodeling in these tumors will be key to dissecting the contribution of individual cell types and matrix components to the broader network. In particular, robust models of the squamous NSCLC subtype and of metastatic processes in NSCLC more generally are currently lacking. Importantly, directly comparing these models and the genomic features associated with them will clarify the context-dependent cues that are currently preventing the development of an integrated model of these processes. It is not currently clear how quickly the ECM is remodeled within the healthy or diseased lung, how long the remodeled matrix persists in a given state, or the temporal hierarchy of how these ECM features evolve over time. An understanding of how early ECM remodeling occurs in the initiation of lung tumors, and whether this tumor-associated ECM remodeling persists following remission will be fundamental to understanding the temporal dynamics of lung tumor progression and relapse.

Our current understanding has identified the impact of ECM composition at the level of individual ECM components. However, the ECM acts a three-dimensional network of matrisomal proteins together with matrisome-associated factors and signaling effectors. An improved understanding of the role of the ECM in regulating dormancy and outgrowth will rely on clearly defining the protein-protein and protein-carbohydrate interactions that form these networks and how individual nodes manipulate the collective three-dimensional architecture of the ECM. High-resolution optical imaging and spatial proteomic technologies to map these interactions will be invaluable in defining these spatial relationships.

Historically, the late presentation of NSCLC has obscured our understanding of early stage disease processes. Furthermore, there is currently no way to establish when tumor cell dissemination establishes intrapulmonary and distant dormant micrometastases to facilitate the direct study of the ECM in these events. However, improved early detection strategies are increasing the proportion of patients diagnosed with early stage disease, and thereby providing opportunities to gain insight into these early dynamics. The analysis of these early stage tumors using improved sequencing and proteomic technologies will also shed light on the true incidence and clonal evolution of the primary and metastatic tumors. Accurate determination of the incidence of intrapulmonary metastases and the contribution of ECM-driven processes to recurrence following surgery will be fundamental to improving patient outcome in early stage disease. Similarly, an understanding of how extracellular matrix remodeling in chronic lung diseases such as chronic obstructive pulmonary disorder and idiopathic pulmonary fibrosis affects the transformation of lung epithelia and the progression of established NSCLC tumors may partly explain why these conditions are associated with an elevated risk of developing these tumors. Inhibition or reversal of ECM remodeling in these fibrotic diseases may be a crucially important approach to prevent lung cancer initiation in these patients.

Taken together, these developments in understanding the structural and functional role of the ECM in NSCLC progression will reveal novel potential therapeutic strategies that are able to identify patients at high risk of developing recurrent disease as well as the optimal therapeutic approaches based on both the cell-intrinsic and -extrinsic mechanisms operating within the tumor microenvironmental rather than focusing on cell-intrinsic somatic genomic alterations alone. Novel therapeutic approaches that sustain disseminated cells in a dormant state, or awaken and eradicate the residual disease, will see lung cancer become a manageable chronic illness and ultimately a curable condition.

## Author Contributions

AP and TC contributed equally to the conception, writing, and reviewing of this manuscript. Both authors contributed to the article and approved the submitted version.

## Conflict of Interest

The authors declare that the research was conducted in the absence of any commercial or financial relationships that could be construed as a potential conflict of interest.

## Abbreviations

ADAMTS: A Disintegrin and Metalloproteinase with Thrombospondin motifs
Akt: v-akt murine Thymoma Viral Oncogene Homolog
AMPK: AMP-activated protein kinas
AXL: AXL Receptor Tyrosine Kinase
BH3: BCL2 Homology Region 3
BMP: bone morphogenetic protein
CD44: cluster of differentiation 44
CDC42: cell division control protein 44 homolog
COL2A1: collagen type II alpha chain
COL5A2: collagen type V alpha chain 2
COPD: Chronic Obstructive Pulmonary Disorder
CXCR1: C-X -C motif chemokine receptor 1
DKK1: Dickopf WNT signaling pathway inhibitor 1
ECM: extracellular matrix
EGF: epidermal growth factor
EGFR: epidermal growth factor receptor
ERK: extracellular signal-regulated kinase
FACIT: fibril associated collagens with interrupted triple helices
FAK: focal adhesion kinase
FGF: fibroblast growth factor
FGF2: fibroblast growth factor 2
GAS6: growth arrest -specific protein 6
HAPLN1: Hyaluronan and Proteoglycan link protein 1
HB-EGF: Heparin-binding EGF-like growth factor
HGF: hepatocyte growth factor
HIF1a: hypoxia inducible factor 1
HOXB9: homeobox protein B9
IL6: interleukin 6
IPF: idiopathic pulmonary fibrosis
JAK2: Janus Kinase 2
JNK: c-JunN-terminal kinase
LEF1: lymphoid enhancer-binding factor 1
LKB1/STK11: liver kinase B1 or serine/threonine kinase 11
LOX: lysyl oxidase
LOXL2: lysyl oxidase like-2
MEK: mitogen activated protein kinase kinase
MIF: macrophage migration inhibitory factor
MLC: myosin light chain
MLCK: myosin light chain kinase
MMP9: matrix metalloproteinase 9
MSI1: RNA-binding protein Musashi homolog 1
mTOR: mammalian target of rapamycin
NK: natural killer cell
NSCLC: non-small cell lung cancer
PDGF: platelet derived growth factor
PET: positron emission tracer
PGP: proline-glycine-proline tripeptide
PI3K: phosphatidylinositol 3-kinase
Raf: rapidly accelerated fibrosarcoma
ROCK: rho-associated coiled -coil kinases
SOX2: SY-box transcription factor 2
STAT5: signal transducer and activator of transcription 5
TAZ: tafazzin
TGFb: transforming growth factor beta
TLR: toll like receptor
TNFa: tumor necrosis factor alpha
uPA: Urokinase-type plasminogen activator
VCAM-1: vascular cell adhesion protein 1
VEGF: vascular endothelial growth factor.
